# Clathrin Light Chains: Not to Be Taken so Lightly

**DOI:** 10.3389/fcell.2021.774587

**Published:** 2021-12-14

**Authors:** Jyoti Das, Mahak Tiwari, Deepa Subramanyam

**Affiliations:** ^1^ National Centre for Cell Science, Pune, India; ^2^ Savitribai Phule Pune University, Pune, India

**Keywords:** clathrin, membrane trafficking, endocytosis, triskelion, physiology, actin

## Abstract

Clathrin is a cytosolic protein involved in the intracellular trafficking of a wide range of cargo. It is composed of three heavy chains and three light chains that together form a triskelion, the subunit that polymerizes to form a clathrin coated vesicle. In addition to its role in membrane trafficking, clathrin is also involved in various cellular and biological processes such as chromosomal segregation during mitosis and organelle biogenesis. Although the role of the heavy chains in regulating important physiological processes has been well documented, we still lack a complete understanding of how clathrin light chains regulate membrane traffic and cell signaling. This review highlights the importance and contributions of clathrin light chains in regulating clathrin assembly, vesicle formation, endocytosis of selective receptors and physiological and developmental processes.

## Introduction

Endocytosis is a process carried out by eukaryotic cells to internalize extracellular molecules, plasma membrane proteins and lipids (Doherty and McMahon, 2009). While several other pathways for endocytosis such as caveolin-mediated endocytosis, phagocytosis and macropinocytosis have been described, clathrin mediated endocytosis (CME) remains the major route for internalization of many membrane lipids and proteins (Kaksonen and Roux, 2018).

CME was first observed by Roth and Porter in 1964 where they found uptake of yolk-containing bristled-coated pits in the mosquito oocyte (Roth and Porter, 1964). Later these bristled-coated structures isolated from pig brain were identified as coat proteins and named ‘Clathrin’ by Barbara Pearse (1975, 1976). Since then, this process has been extensively studied and although we have a fairly good understanding of the process itself, many unanswered questions still remain about how over 50 molecules that take part in this molecular process (Haucke and Kozlov, 2018), come together in a highly coordinated manner.

CME is characterized by the recruitment of clathrin and its associated molecules to the plasma membrane allowing the formation of clathrin-coated vesicles (CCVs). The formation of CCVs involves the polymerization of ‘clathrin triskelia’, which are the basic building blocks of the clathrin coats (Kaksonen and Roux, 2018). A triskelion is composed of three clathrin heavy chains (CHC) (∼190 kDa) each of which is associated with a smaller clathrin light chain (CLC) (∼25 kDa). While the major role of the clathrin heavy chain is in intracellular trafficking, it is also involved in several other processes including chromosomal segregation during mitosis (Royle et al., 2005), regulation of basal NF-κB activity in epithelial cells (Kim et al., 2011), control of neuropeptide degradation and secretion during neuronal development (Nahorski et al., 2018), and maintenance of mouse embryonic stem cell pluripotency (Narayana et al., 2019; Mote et al., 2020).

Variations in the clathrin heavy and light chains alter the biophysical properties of the clathrin lattice, in turn affecting trafficking of receptors and thereby several physiological functions of the cell. The heavy chain is essential for triskelion assembly and for all clathrin-dependent endocytic events, with a number of excellent reviews highlighting the function of this protein (Kirchhausen, 2000; Brodsky, 2012; Kirchhausen et al., 2014; Brodsky, 2016; Kaksonen and Roux, 2018; Briant et al., 2020). In contrast, the role of the clathrin light chains remains relatively under-explored. In this review, we look at how the clathrin light chains affect clathrin polymerization, vesicle formation, receptor trafficking and cell signaling.

## Clathrin Genes and Proteins

In metazoans, the clathrin heavy chain protein is encoded by a single gene, *Cltc*. In humans, due to large-scale gene duplications during chordate evolution, there are two CHC paralogs, CHC17 (encoded by *Cltc*) and CHC22 (encoded by *Cltcl1*) based on their location on chromosome 17 and 22, respectively. Although *Cltcl1* is found in several other vertebrate species, it is functional only in humans. In mice, only a pseudogene for CHC22 is present (Wakeham et al., 2005). In yeast and invertebrates such as *Drosophila* and *Caenorhabditis elegans*, the CHC protein is encoded by a single gene. Plants have two genes for the clathrin heavy chain, CHC1 and CHC2 (Baisa et al., 2013).

In invertebrates, the clathrin light chain is encoded by a single gene. However, as a result of local gene duplication, higher eukaryotes have two light chains, CLCa and CLCb encoded by the genes *Clta* and *Cltb*, respectively (Wakeham et al., 2005). They both share 60% homology in their amino acid sequence but are expressed at different levels in various vertebrate tissues (Wu et al., 2016). Despite having considerable divergence in sequence, the single light chain from yeast shares various physical properties with mammalian light chains (Silveira et al., 1990). In plants, the three clathrin light chain genes CLC1, CLC2 and CLC3 (Scheele and Holstein, 2002; Baisa et al., 2013) share at least 30% sequence homology with mammalian CLCs (Wang et al., 2013).

## Clathrin Light Chain Domain Organization and Function

**FIGURE 1:**
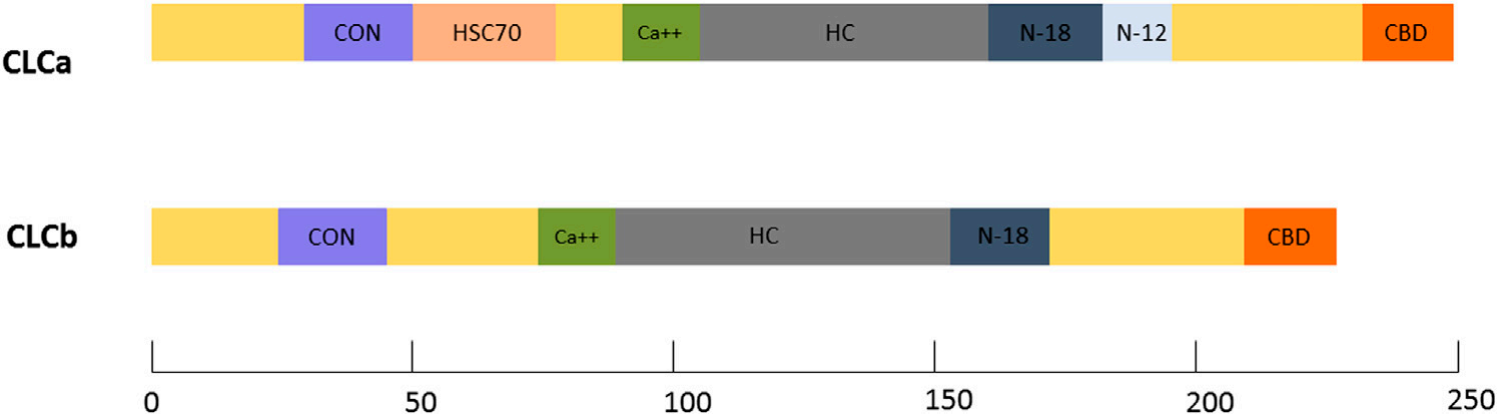
CLC protein domains: Domain maps of the vertebrate CLCs, CLCa and CLCb. Common functional domains indicated include the consensus sequence (CON) shared by all vertebrate CLCs, the calcium-binding sequence (Ca++), the heavy chain-binding region (HC), the neuronal inserts of 18(N-18) and 12(N-12) residues, and the calmodulin-binding domain (CBD). Unique to CLCa is a region that can stimulate the uncoating ATPase, HSC70, *in vitro*.

Vertebrate CLCs contain a consensus region of 22 amino acids shared by both CLCa and CLCb. Additionally, they also include distinct domains for binding to calcium, clathrin heavy chain, calmodulin and a neuron-specific insertion sequence (Brodsky, 2012). CLCa contains a unique Hsc70 binding region (DeLuca-Flaherty et al., 1990). However, functions have been attributed to only some of these domains. A detailed representation of CLC domain organization can be found in Figure 1.

In mammals, at the N-terminus, a 22 amino acid conserved sequence is shared by CLCa (residues 28–49) and CLCb (residues 20–41) with the negatively charged residues, EED responsible for CLC binding to the CHC knee (Brodsky, 2012). This conserved sequence is also the binding site for the Huntington interacting protein (HIP) family (Chen and Brodsky, 2005), and plays a role in regulating clathrin self-assembly (Legendre-Guillemin et al., 2005; Ybe et al., 2007a).

CLCa has an Hsc70 binding sequence that was shown to stimulate uncoating *in vitro* (DeLuca-Flaherty et al., 1990). However later studies suggested that uncoating of vesicles could also be done in the absence of CLCs *in vitro* (Ungewickell et al., 1995) Both the light chains also have a calcium binding region (Nathke et al., 1990) and calmodulin binding domain (Pley et al., 1995) present at the centre and C-terminal, respectively. While these domains have been found to play a role in *in vitro* studies, no function has been attributed to them *in vivo*.

In *Dictyostelium*, overexpression of the C-terminal fragment of CLCa in *clc* null cells produced dynamic punctae distribution along the plasma membrane and within the cytoplasm, similar to full-length CLCa (Wang et al., 2006), indicating that its function can be attributed almost entirely to the C-terminal domain.

*In vitro* assembly of the clathrin hub, and CHC trimer stability is enhanced by the C-terminal domain of the light chain (Ybe et al., 2007b). Co-expressing the trimer-defective hub heavy chain mutant C1573A, along with the light chain C-terminal domain construct could achieve approximately 67% of wild-type clathrin assembly.

## Splice Variants and Insertion Sequences in Light Chains

CLCs undergo alternate mRNA splicing in vertebrates, giving rise to four isoforms for CLCa and two for CLCb (Blue et al., 2018a). Exons 5 and 6, encoding 18 and 12 amino acids respectively, in the *Clta* gene are alternatively spliced resulting in four isoforms. These are: 1) neuronal CLCa (nCLCa) containing both 18 and 12 amino acid residue insertions; 2) a splice variant containing only the 18 residue-insertion found only in brain; 3) a splice variant containing only the 12 residue-insertion found in brain, heart and skeletal muscle; and 4) a splice variant without either insertion.

In vertebrates, the two splice variants for CLCb include an isoform having an insert of 18 residues present in neurons (nCLCb) and another that lacks the insert in non-neuronal tissues (Wong et al., 1990; Blue et al., 2018a). In rats, the CLCb gene contains six exons. The isoform containing all exons is brain-specific (LCB2), while the isoform lacking exon 5 (LCB3) is present in other tissues. LCB2 is predominantly present in primary rat neuronal cultures, whereas LCB3 is present in primary rat glial cultures (Stamm et al., 1992).

Due to the insertions mentioned above, neuronal splice variants have a higher molecular mass than CLCs in other cell types. Under oxidizing conditions, CLC isoforms can form internal disulfide bonds. Both brain-specific CLCa isoforms, contain the 12 residue insert in exon 5, allowing the formation of internal disulfide bonds between two cysteine residues *in vitro*, while the smallest CLCa isoform only has a single cysteine residue. Both CLCb isoforms have two cysteine residues present at the C-terminal. *In vitro* purification of CLCs in the presence or absence of thiols or alkylating agents caused an alteration in electrophoretic mobility depending on the formation of disulfide bonds (Parham et al., 1989). The electrophoretic mobility change of CLCs was also found to be species- and tissue-specific. This could be due to the presence of different isoforms (Ungewickell, 1983), disulfide bond formation (Parham et al., 1989), or other post-translational modifications such as phosphorylation (Ferreira et al., 2012).

At all developmental stages, the Clta transcript in the mouse heart does not include exon 5. However, due to alternative splicing, Clta exon 6 is included at a low level at birth with its inclusion increasing in adulthood (Giudice et al., 2014; Blue et al., 2018b). This suggests that expression of CLC splice variants is variable and can change with the stage of development, and in a tissue-specific manner.

Both the CLCs are developmentally and tissue-specifically regulated by alternative splicing but the physiological and functional implications of different splice variants of CLCs still remain unexplored. Although we still lack complete understanding of tissue-specific expression patterns of alternatively spliced variants of CLCs, one can speculate that the presence or absence of particular exons may lead to a change in interacting partners thereby influencing function.

Despite having 60% sequence similarity, the two light chains are diverse in nature due to alternative splicing, internal disulfide bond formation and tissue specific expression patterns, allowing speculation that their ability to perform distinct physiological and functional roles could be attributed to such differences.

## Phosphorylation of Clathrin Light Chains

The phosphorylation of clathrin light chains was initially identified *in vitro* in coated vesicles isolated from bovine brains (Usami et al., 1985). Using rat reticulocytes, Bar-Zvi et al. (1998), then demonstrated that unassembled pools of CLCb were highly phosphorylated. Further investigations revealed that CLCb underwent phosphorylation mediated by Casein Kinase 2 at the N-terminal serine residues at positions 11 and 13. These residues are unique to CLCb and absent in CLCa (Hill et al., 1988). However both CLCa and CLCb contain the phosphorylation site for G-Protein coupled receptor kinase 2 (GRK2) at Ser204. Mutation of all phosphorylation sites in CLCb impeded internalization of purinergic GPCRs, P2Y_1_ and P2Y_12_, with phosphorylation of Ser204 being specific for P2Y_12_ uptake (Ferreira et al., 2012). Phosphorylation of CLCb at Ser204 was also required for lattice rearrangement and curvature generation by regulating clathrin exchange in a cargo-dependent manner (Maib et al., 2018). Together these findings indicate a role for phosphorylated forms of clathrin light chains in regulating the uptake of specific membrane-resident proteins.

## Interaction With the Clathrin Heavy Chain

Both CLCs can bind and regulate CHC17, but do not functionally interact with CHC22 (Towler et al., 2004). Previous studies have shown that CLCs bind to the proximal leg of the heavy chain via their central region (Kirchhausen et al., 1983; Brodsky et al., 1987; Jackson et al., 1987; Nathke et al., 1992; Liu et al., 1995). Using a yeast-two hybrid system it was shown that the core interaction occurs between CHC residues 1,267–1,522, and CLCb residues 90–157 (Chen et al., 2002). Mutations in the central region (residues 90–157) of CLCb disrupt the alpha-helical structure suggesting that this region is crucial for interaction with CHC. Cryo-EM based structural analysis revealed that two tryptophan residues (W105 and W127) were required for light chain binding to the heavy chain. Mutation of W105 to arginine disrupted CLC-CHC binding, but could be rescued by mutation of lysine to glutamate at residue position 1,326 of the heavy chain. Additionally, two helices present in the CLC trimerization domain (TxD) tended to form stable association with two heavy chain TxDs in trans conformation, connecting adjacent legs and forming the triskelion vertex (Morris et al., 2019).

## Role of Light Chains in Clathrin Assembly and Disassembly

### Assembly and Stabilization of the Clathrin Triskelion

Studies done in yeast suggest that light chains affect the trimerization and stability of the heavy chain (Silveira et al., 1990; Huang et al., 1997). The amount of heavy chain in light chain-deficient strains is reduced to 20–25% of their wild-type counterparts, most of which are not trimerized (Huang et al., 1997). CLC-deficient strains have also been known to show a slow-growth phenotype, similar to CHC deficient strains (Silveira et al., 1990), indicative of the fact that the light chain in yeast is essential for heavy chain trimerization and stability. In *Dictyostelium* however, the light chains do not contribute to heavy chain trimerization or stability, but affect the assembly of triskelia onto intracellular membranes (Wang et al., 2003). This indicates a species-specific role for light chains in conferring stability to triskelia. The reason behind this is not completely understood as the domain structure of CLCs across species remains conserved, despite having little similarity in amino acid sequence (Wang et al., 2003).

CLCs stabilize the triskelion via their C-terminal region which interacts with the vertex and reduces the flexibility of the legs to produce triskelia with uniform vertex geometry (Ybe et al., 2007b). In the absence of light chains, the legs can adopt various geometries due to increased flexibility at the vertex (Ybe et al., 2007b). These changes in triskelion structure can affect cage and lattice forming properties of clathrin, which is discussed in the next section.

### Assembly of Clathrin Cages and Lattices

The role of clathrin light chains in cage assembly and disassembly has been studied extensively since their identification in 1981. Early studies reported that treatment of clathrin with elastase, which selectively digests the light chains, renders the triskelion incapable of correctly assembling and forming cages (Kirchhausen and Harrison, 1981; Schmid et al., 1982). However, it was later shown that heavy chain trimers can reassemble into polygonal cages even in the absence of light chains (Winkler and Stanley, 1983). We now know that the light chains function as negative regulators of cage assembly, as shown by the following studies.

*In vitro* studies using recombinant hubs (trimeric clathrin heavy chain structures without the distal domain and the N-terminal region) have shown that while hubs lacking light chains can self-assemble reversibly at a physiological pH, they can self-assemble only at a pH below 6.5 in the presence of light chains (Liu et al., 1995). They also require the presence of adaptor proteins such as AP-1, AP-2 or the neuron-specific AP-180 to assemble at physiological pH (Ahle and Ungewickell, 1986; Keen, 1990; Pearse and Robinson, 1990; Lindner and Ungewickell, 1992). These reports show that light chains regulate cage assembly by preventing unnecessary polymerization of clathrin triskelia and allowing regulated assembly by adaptor molecules.

Light chains have a negatively charged EED domain which can bind to the positively charged KR loop present in the crease of the heavy chain (Wilbur et al., 2010). As mentioned earlier, this interaction influences the flexibility at the knee, which affects lattice assembly. If CLC is bound, the knee is straight and the triskelion is more rigid. Such a conformation inhibits cage assembly. The retraction of light chain produces more compact and flexible triskelia and allows the triskelia to form clathrin cages (Wilbur et al., 2010).

Adaptors can overcome the effect of light chains by introducing competing positively charged residues that can free up the heavy chain to polymerize (Greene et al., 2000). AP-2 can directly bind to the clathrin heavy chain (Owen et al., 2000). By aligning the distal regions of the heavy chain with the proximal hub segments it provides the competing residues required to reverse the effect of light chains (Greene et al., 2000). It has recently been shown that these interactions between adaptors and the clathrin coat also regulate cargo binding and coat curvature, by reconfiguring low-affinity, high-avidity interactions (Kovtun et al., 2020).

The presence of light chains also increases the stiffness of clathrin lattices which increases the ability of clathrin to deform liposomal membranes into buds (Dannhauser et al., 2015). Budding efficiency has also been shown to vary with different CLC isoforms. Lattices containing neuronal isoforms of the light chains exhibit a poorer lattice quality and a lower budding efficiency compared to lattices with non-neuronal isoforms (Redlingshöfer et al., 2020).

### Disassembly of the Clathrin Cage

Hsc70, like most chaperone proteins, requires cofactors to recruit the chaperone to the target site (Böcking et al., 2011). Auxilin 1 and GAK (also known as Auxilin 2) are two cofactors of Hsc70 belonging to the DnaJ family of chaperones (Jiang et al., 1997; Umeda et al., 2000). While Auxilin 1 is expressed only in the brain (Böcking et al., 2011), GAK is expressed in several other tissues (Kanaoka et al., 1997; Kimura et al., 1997). Whether the light chains directly affect Auxilin and GAK mediated uncoating is still unclear. According to Ungewickell et al. (1995), CLCs are dispensable for Auxilin-mediated uncoating of clathrin-coated vesicles. Later studies suggest that although the light chains are not essential for uncoating, their removal significantly reduces the efficiency with which Auxilin facilitates disassembly (Young et al., 2013). A study by Ferreira et al. (2012) suggests that clathrin light chain B can modulate the interaction between auxilin and clathrin heavy chain, thereby regulating the process of vesicle uncoating.

## Regulation of Receptor Trafficking by Clathrin Light Chains

The physiological importance of light chains has mostly been studied using knockdown or knockouts of the light chains, or through the use of mutant forms to study receptor trafficking (Huang et al., 2004; Poupon et al., 2008; Majeed et al., 2014; Wu et al., 2016; Redlingshöfer et al., 2020). Knockout of CLCa in mice hampered the internalization of Transforming growth factor β receptor2 (TGFβR2), affecting antibody isotype switching in B lymphocytes (Wu et al., 2016). Knockdown of both light chains in mammalian HeLa cells did not affect the internalization of β1 integrin but disrupted its recycling back to the plasma membrane (Majeed et al., 2014). CLC knockdown (KD) also altered the targeting of cation-independent mannose-6 phosphate receptor (CI-MPR) to the endosome, resulting in clustering of the receptor near the trans-Golgi network, leading to a delay in processing of the lysosomal hydrolase cathepsin D in HeLa and Cos7 cells (Poupon et al., 2008).

Internalization of GPCRs was also shown to be dependent on CLCb phosphorylation (Ferreira et al., 2012). Internalization of P2Y_12_ receptor, a member of a family of purinergic GPCRs, in 1321N1 astrocytoma cells is regulated by phosphorylation of CLCb. Trafficking of low-density lipoprotein on the other hand, is not affected by the removal of light chains (Poupon et al., 2008). Furthermore, internalization of EGFR was also not affected by siRNA-mediated knockdown of both the light chains in HeLa cells (Huang et al., 2004). However, a study using single light chain-expressing H1299 cells, a non-small cell lung cancer cell line, showed accelerated internalization of EGFR in cells that expressed only CLCb in contrast to wild type and CLCa-only expressing cells (Chen et al., 2017). Similar observations have been made with respect to internalization of transferrin receptor (Tfr) (Huang et al., 2004; Chen et al., 2017). Tfr internalization can also be dependent on the phosphorylation status of CLCb. When transferrin receptor is clustered with other cargo, its uptake can become sensitive to the status of CLCb phosphorylation. In 1321N1 human astrocytoma cells, packaging of Tfr with P2Y_12_ receptor resulted in delayed internalization of Tfr in presence of a phosphorylation-deficient mutant of CLCb (Maib et al., 2018), with similar results also observed in HeLa cells. Knockdown of both the light chains attenuated Tfr recycling in HeLa cells (Majeed et al., 2014), which remains unaffected in single light chain expressing H1299 cells (Chen et al., 2017).

From these studies one can infer that i) dependence of receptor trafficking on light chains is influenced by other factors such as the cell type and presence of other cargo; ii) CLCa and CLCb can differentially affect cargo uptake; and iii) phosphorylation status of CLCb can potentially be a method of regulating receptor trafficking.

## Physiological Significance of Clathrin Light Chains

Altered trafficking of receptors can compromise cell signaling, which is an important regulator of several physiological functions. The sections below discuss how light chains regulate important biological functions and pathological conditions. These are also summarized in Table 1.

**TABLE 1:** Table showing the role of clathrin light chains in various physiological processes.

Physiological process	Cell type/Model organism	Method of study	Observations/Inference	References
Development	Mice	CLCa Knockout	CLCa is essential for B-cell development and antibody production	Wu et al. (2016)
	*Drosophila melanogaster*	CLC Knockdown with overexpression of Rac1	CLCs present on the endosomes bind LRRK2 to inhibit Rac1 activation. This interaction is necessary for *Drosophila* eye development	Schreij et al. (2015)
	*Arabidopsis thaliana*	T-DNA insertion lines for CLC1, CLC2 and CLC3. CLC2 and CL3 double mutant line	CLC2 and CLC3 are necessary for auxin regulation of plant development. CLC1 is essential to maintain gamete viability	Wang et al. (2013)
	*Dictyostelium*	CLC knockout	CLC is required for formation of fruiting bodies	Wang et al. (2003)
Cell spreading and migration	HeLa and H1299 cells	CLCa and CLCb knockdown	CLCs are required for β1 integrin dependant cell migration	Majeed et al. (2014)
	HeLa, H1299 and HEK293T cells	CLCa and CLCb knockdown	CLCa and not CLCb is required for Focal adhesion maturation, and consequently cell spreading and migration	Tsygankova and Keen (2019)
	U373 astrocytes	Overexpression of a dominant negative CLCb	CLCb is involved in motility of astrocytes	Saffarian et al. (2009)
	HEK293T cells	Deletion of CLCa and CLCb	CLCs are required for invadopodia formation	Mukenhirn et al. (2021)
Neuronal function and neurodegeneration	*Drosophila melanogaster*	Photo-inactivation of the dmCLC	CLC is required for synaptic vesicle re-formation	Heerssen et al. (2008)
	Mice	CLCa and CLCb knockout	CLCs have distinct roles in synaptic vesicle recycling	Redlingshöfer et al. (2020)
	Patients with Alzheimer’s disease	IHC studies in hippocampal tissues of patients with AD	Decrease in levels of CLCb at the synapse in AD patients indicating hampered clathrin transport	Nakamura et al. (1994a)
	Patients with Pick’s disease	IHC Studies in hippocampal tissues of patients with Pick’s disease	Abnormal levels of CLCs in neuronal perikarya of Pick’s disease patients	Nakamura et al. (1994b)
	Alzheimer’s disease mice models	Proteomic analysis of the hippocampus of the Alzheimer’s disease mice models	Upregulated CLCb levels in the hippocampus of AD mice	Takano et al. (2013)
Cell division	*Arabidopsis thaliana*	Overexpression of CLC Fused to mGFP5, mOrange or enhanced cyan fluorescent protein (eCFP)	CLC associates with the distal plasma membrane of expanding root hairs	Konopka et al. (2008)
	U2OS cells	Overexpression of GFP-Clta and mRFP-MAD2B	CLC associates with MAD2B at the mitotic spindle during mitosis	Medendorp et al. (2010)

### Development

Mammalian development is dependent on the presence and action of light chains, especially in the context of B-cell development (Wu et al., 2016). The lymphoid tissue shows an almost exclusive expression of CLCa. Germinal centres in CLCa knockout mice have fewer B cells, which predominantly produce IgA antibodies. This increased IgA production is attributed to enhanced signaling by the TGFβR2 receptor due to its defective endocytosis (Wu et al., 2016).

Normal eye development in *Drosophila* is dependent on clathrin light chains (Schreij et al., 2015). LRRK2, a high molecular weight Ras GTPase, directly binds to light chains present on the endosomes. CLC and LRRK2 interact to inhibit Rac1 activation, with disruption in this pathway resulting in altered eye development in *Drosophila* (Schreij et al., 2015).

Clathrin light chains also play an important role in plant development. The loss of light chains, CLC2 and CLC3 affect auxin-regulated endocytosis, resulting in multiple developmental defects in *Arabidopsis thaliana* (Wang et al., 2013). Additionally CLC1 mutant pollen also display reduced viability (Wang et al., 2013), suggesting that the three light chains have specific and independent roles in gamete formation and development, and that the loss of a single light chain may not be compensated for by the presence of the other two.

*Dictyostelium* CLC null-mutants show defects in development as demonstrated by their inability to form fruiting bodies (Wang et al., 2003). Overexpression of the C-terminal domain of CLC rescues this phenotype with robust fruiting body formation indistinguishable from wild type fruiting bodies (Wang et al., 2006). Loss of CLC results in larger vacuoles in *Dictyostelium*, indicative of disruption of osmoregulation (Stavrou and O’Halloran, 2006). Together, these studies from different species indicate that clathrin light chains perform distinct and diverse functions during development.

### Cell Spreading and Migration

As mentioned above, depletion of both the light chains reduced the surface expression of β1 integrin due to altered recycling, which decreased cell migration in both HeLa and H1299 cells (Majeed et al., 2014). Migratory displacement of HeLa cells was reduced by 22% in contrast to H1299 cells whose displacement was reduced by 41% upon loss of light chains (Majeed et al., 2014). Non-small cell lung cancers expressed elevated levels of CLCb resulting in increased activation of Dynamin1 via a pathway involving Akt/GSK3β phosphorylation. This resulted in abnormal EGFR trafficking and signaling, leading to increased migration and metastasis (Chen et al., 2017). Another recent study showed that CLCa and not CLCb was important for focal adhesion (FA) maturation, cell spreading and migration, with CLCa targeting FAKs to nascent FAs. In the absence of CLCa these transient nascent structures were unable to mature to radially elongated FAs due to reduction in integrin-mediated activation of Src and Rac (Tsygankova and Keen, 2019).

In U373 astrocytes, overexpression of a dominant negative CLCb mutant which could bind to the heavy chain but not to Hip1/R, resulted in increased motility due to reduction in plaque formation (Saffarian et al., 2009).

Recently, it has also been shown that light chains are involved in invadopodia formation in HEK293T cells (Mukenhirn et al., 2021). Deletion of both light chains resulted in increased recycling of MMP14, a matrix metalloproteinase protein whose increased surface expression has been known to coincide with malignant cancer progression. Furthermore, loss of the light chains caused actin to polymerize and form patches on the plasma membrane. These actin structures along with MMP14 clusters on the plasma membrane formed mature invadopodia. Invadopodia are important for embryonic development, bone remodeling and cancer metastasis (Mukenhirn et al., 2021). Altered invadopodia formation could possibly affect these important physiological processes.

Together these studies demonstrate that individual clathrin light chains regulate migration and invasion differentially depending on the cell type. Additionally, these phenotypes may also be a reflection of their interaction with specific proteins that are also expressed in a cell-type dependent manner.

### Neuronal Function and Neurodegeneration

Besides playing an important role in cell-signaling by regulating receptor trafficking, clathrin also plays an important role in neuron-specific functions such as synaptic vesicle recycling and neurotransmitter receptor trafficking. Photo-inactivation of the clathrin light chain in *Drosophila* at neuromuscular junctions (NMJ) resulted in a block in synaptic vesicle re-formation. Although clathrin-independent mechanisms of membrane internalization do exist at the *Drosophila* NMJ, these were unable to generate fusion-competent vesicles, indicating a specificity for the light chain in this context (Heerssen et al., 2008). A similar phenotype was also seen in CLCa and CLCb knockout mice. Knockout of individual light chains in mice showed electrophysiological defects, indicative of impaired synaptic vesicle recycling (Redlingshöfer et al., 2020). Interestingly, CLCa and CLCb knockout mice exhibited different phenotypes. In the synapses of cerebellar neurons, CLCa knockout mice showed reduced number of synaptic vesicles whereas CLCb knockout mice did not show any decrease compared to wild type mice. However, in hippocampal neurons, CLCa knockout mice showed a decrease in the number of synaptic vesicles, while CLCb knockout mice showed almost twice the number of vesicles relative to wild type mice. CLCa knockout mice also showed defects in motor function (Redlingshöfer et al., 2020). This highlights the fact that CLCa and CLCb have distinct roles in synaptic vesicle recycling and also indicates that the same paralog can differentially affect function in neurons from different regions of the brain.

Altered endocytosis is also associated with several neurodegenerative disorders. Immunohistochemical analysis of the hippocampus from individuals with Alzheimer’s disease show an abnormal distribution of clathrin light chains, with a high concentration of CLCb detected in neurofibrillary tangles (Nakamura et al., 1994a). Under normal circumstances, clathrin is concentrated at the synaptic terminals. However, in patients with Alzheimer’s disease, CLCb is reduced at the synapse indicating that the normal transport of clathrin from the neuronal perikarya to the axon terminals is hampered (Nakamura et al., 1994a). The implications, if any, of this abnormal distribution of light chains are still not understood. Proteomic analysis of the hippocampus of the Alzheimer’s disease mice models showed that CLCb and Dynamin 1 were upregulated in diseased mice compared to wild type mice. Interestingly, no significant difference was observed in the expression of CLCa (Takano et al., 2013). Abnormal distribution of clathrin light chains was also observed in the brains of patients with Pick’s disease. Immunohistochemical analysis showed a high concentration of light chains in Pick’s bodies. In neurons of the dentate gyrus of Pick’s disease patients, light chains were also found in increased amounts in the neuronal perikarya compared to healthy individuals (Nakamura et al., 1994b). While the cause and implications of the increase in CLCs are still not understood, one can speculate that the altered expression and localization may contribute to the disease phenotype.

### Cell Division

Clathrin-mediated endocytosis is a continuous event in non-dividing cells. However, in cells undergoing mitosis, endocytic events stop (Fielding et al., 2012), and clathrin accumulates at the spindle apparatus carrying out an important function, independent of trafficking (Royle, 2012). It functions by crosslinking the microtubules of the kinetochore to stabilize the mitotic spindle (Royle et al., 2005). CHC also promotes centrosome maturation by stabilizing the microtubule-binding protein ch-TOG (colonic, hepatic tumor overexpressed gene) (Foraker et al., 2012).

A study in *Arabidopsis thaliana* has shown that CLCs accumulate at the mitotic spindle during cell division. GFP-tagged CLC has been shown to be associated with the distal plasma membrane in expanding root hairs, and at the cell plate in dividing root cells (Konopka et al., 2008).

In *Dictyostelium*, CLC null mutants display a defect in cytokinesis, which can be rescued by overexpression of the C-terminal domain-containing CLC construct (residues 124–194) (Wang et al., 2006).

Another important protein involved in mitosis is the mitotic arrest deficient protein, MAD2B which binds to, and inhibits the anaphase promoting complex (APC) (Chen and Fang, 2001). Depletion of MAD2B in renal carcinoma cells caused a significant increase in the number of misaligned chromosomes. MAD2B interacts with the C-terminus of CLCa during the G2/M phase of the cell cycle, with knockdown of MAD2B resulting in redistribution of CLCa away from the mitotic spindle (Medendorp et al., 2010). The functional relevance of this interaction is as yet unexplored. It should also be noted that heavy chain distribution remained unaffected upon depletion of MAD2B (Medendorp et al., 2010).

## Clathrin Light Chains: Connecting the Endocytic Machinery to the Actin Cytoskeleton

The role of actin in endocytosis is well established. Actin is recruited to sites of endocytosis and helps the membrane to invaginate and form coated pits (Engqvist-Goldstein and Drubin, 2003; Mooren et al., 2012). Clathrin light chains can bind to Hip1/R proteins through a conserved domain present at their N-terminus, which in turn binds to actin (Chen and Brodsky, 2005). The light chains therefore act as a connecting link between the endocytic machinery and the cytoskeleton.

Hip1/R proteins can bind to actin through their THATCH domain independent of CLCs. Binding of the light chains to the coiled-coil domains of Hip1 and Hip1R reduce their actin-binding activity. This suggests that Hip proteins do not interact with actin while incorporated into the clathrin coat. Instead Hip proteins interact with actin at the neck of the budding vesicle or edge of the clathrin coat, promoting development of a budding vesicle (Wilbur et al., 2008; Boettner et al., 2011). Hip1 binding to CLC is necessary for its targeting to clathrin-coated pits (Legendre-Guillemin et al., 2005) and loss of CLCs result in mislocalization of Hip1R and overassembly of actin patches (Poupon et al., 2008), further emphasizing the point that light chains are essential for recruiting actin to sites of endocytosis by interacting with Hip1/R proteins.

In yeast, all clathrin-dependent endocytic events are actin-dependent and therefore, light chain-dependent (Chu et al., 1996). In mammalian cells however, the light chains and actin are not essential for CME to occur.

In what context is an endocytic event light chain-dependent or -independent? The factor that dictates the requirement of light chains is the amount of force that is required for the membrane to invaginate. Membrane tension opposes membrane deformation. Invagination of membranes with high tension require greater force. Boulant et al. (2011) showed that actin was recruited by the light chains to counteract membrane tension in polarized MDCK cells. It is plausible that in instances where clathrin polymerization does not produce enough energy to bend the membrane, the light chains recruit actin, which polymerizes and provides energy for membrane invagination. Membrane tension may differ between cell types. This explains why the uptake of the same receptor may be light chain dependent in one type of cell, and independent in another. Another factor that opposes membrane budding is turgor pressure. Turgor pressure of yeast is higher than that of mammalian cells (Aghamohammadzadeh and Ayscough, 2009), which may explain why all clathrin-dependent endocytic events in yeast are light chain and actin-dependent (Goode et al., 2015). It is important to note however, that in plants, which have a similarly high turgor pressure as yeast, actin is not required for endocytosis (Baisa et al., 2013), allowing speculation that other proteins may be involved in this process.

**FIGURE 2:**
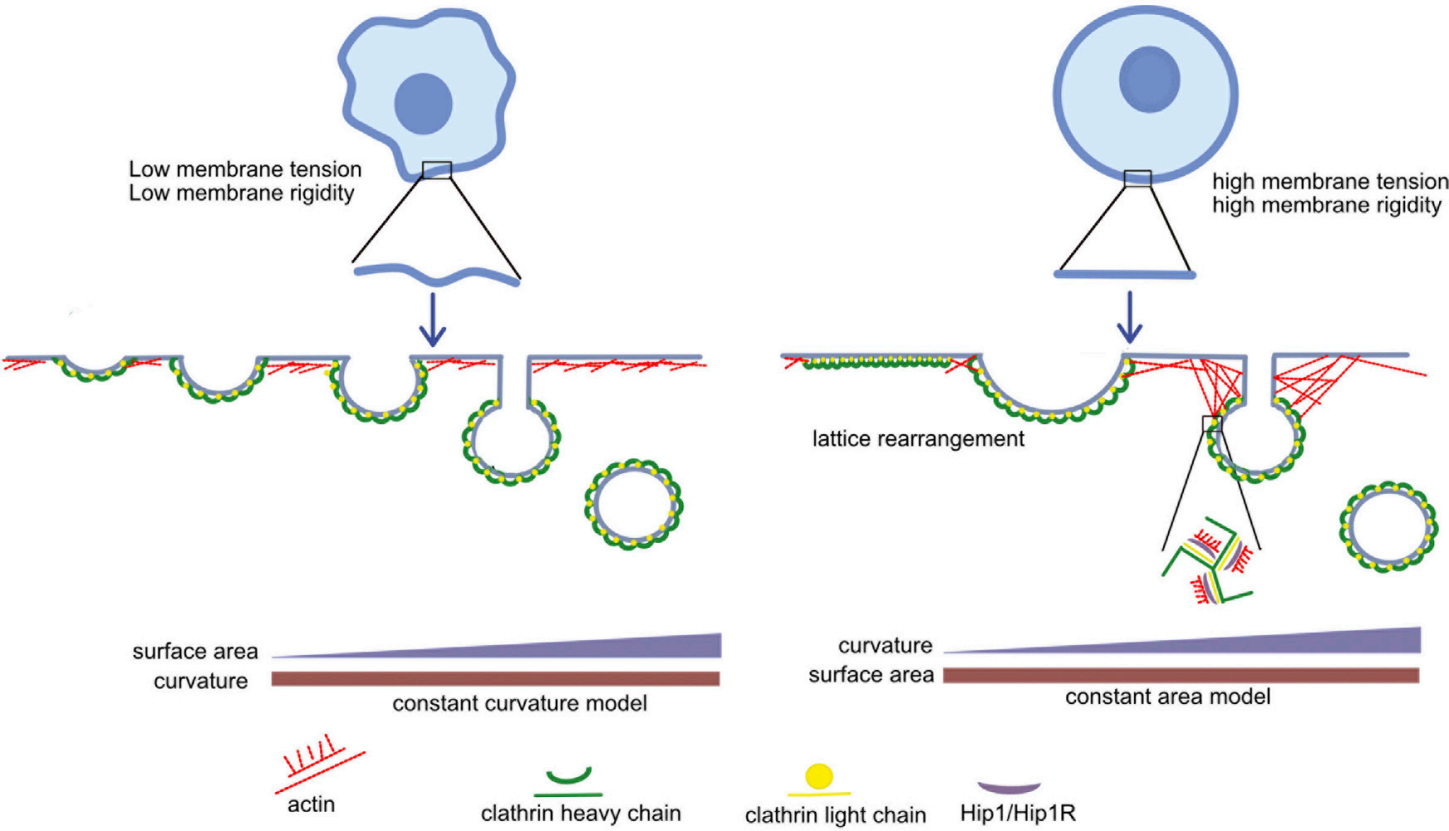
Interaction of clathrin light chains with actin: When forces opposing membrane invagination (such as membrane tension and membrane rigidity) are high, clathrin first assembles as a flat lattice, and light chain-dependent rearrangement takes place to introduce curvature (constant area model). Since the polymerization energy of clathrin is insufficient to deform the membrane, the actin cytoskeleton is recruited by light chains to further counteract these opposing forces. On the other hand, when forces opposing membrane invagination are low, the polymerization energy of clathrin is sufficient to deform the membrane. Clathrin directly polymerizes onto the budding membrane (constant curvature model) and clathrin light chains and the actin cytoskeleton are not required.

Recruiting actin to provide energy for membrane invagination is not the only way light chains help in vesicle formation. As mentioned above, light chains are also involved in lattice rearrangement which introduces membrane curvature in flat clathrin lattices as they transform into shallow pits (Maib et al., 2018). However, clathrin does not always assemble as a flat lattice first. This happens only when the constant area model of membrane invagination is followed. According to this model the clathrin coat assembles into a flat lattice of a given area. The lattice is then remodeled by inserting pentagons to introduce curvature without changing the area. Another proposed model is the constant curvature model for membrane invagination according to which clathrin assembles directly into a bud of constant curvature. As a spherical vesicle is formed from a shallow pit, the clathrin coated area increases (Figure 2). Both these models have been shown to exist *in vitro* (Scott et al., 2018). An increase in membrane tension also increases the number of flat clathrin lattices (Bucher et al., 2018). Based on these studies, Maib et al. (2018) hypothesized that in cases where the polymerization energy of the clathrin triskelia is not sufficient to deform the membrane directly, it will initially assemble as a flat lattice, whereas membranes that are easier to deform might follow the path of constant curvature, directly polymerizing into spherical vesicles.

To summarize, when there is low membrane tension, clathrin may directly polymerize onto the budding surface and light chains will not be required to rearrange the lattice and recruit actin. When there is high membrane tension clathrin may first assemble as a flat lattice which can then be rearranged with the help of light chains and actin to provide energy for membrane invagination.

This ability of the light chains to bind to Hip1/R protein and recruit actin is often exploited by bacteria and viruses to facilitate their entry into cells. *Listeria monocytogenes* for example, binds to cadherin through internalin, a protein which induces phosphorylation of the heavy chain. This phosphorylation recruits actin through the Hip1/R binding domain of the light chains to surround clathrin at the membrane and facilitate the entry of the pathogen into the cell (Bonazzi et al., 2011).

## Conclusions and Future Scope

Five decades of research has provided a huge amount of insight into the complex process of CME (Kaksonen and Roux, 2018; Briant et al., 2020). While studies reveal the role of clathrin light chains in regulating clathrin assembly and several physiological processes, a number of open questions remain unanswered. For example, the specific roles of CLCa and CLCb and their splice variants are not completely understood. While recent studies have shed some light on their specific functions (Wu et al., 2016; Maib et al., 2018; Tsygankova and Keen, 2019; Redlingshöfer et al., 2020), we are only beginning to appreciate the role of each paralog. Apart from this, there is little clarity on why the requirement for light chains is different between different species and cell types. Other questions that need to be answered include the role of light chains in auxilin and GAK-mediated uncoating, mitosis, cell migration and neurodegeneration.

The molecular complexity and dynamic nature of endocytosis make it a difficult process to study. The presence of two paralogs further complicates the problem of elucidating the role of light chains. Small interfering RNA (siRNA)-mediated knockdown of CLCb is often compensated by increased expression of CLCa, whereas knockdown of CLCa is often accompanied by decrease in CHC expression (Majeed et al., 2014). This can lead to inconclusive and confounding results. Use of molecular techniques such as CRISPR-Cas9 genome editing can help overcome these problems by generating single isoform expressing cells. Spatio-temporal deletion of CLCs can further be instrumental in understanding their function in regulating physiological processes.
